# Protocol for ultrafast immunolabeling and 3D imaging of whole organs and large tissues

**DOI:** 10.1016/j.xpro.2026.104623

**Published:** 2026-06-06

**Authors:** Zhangfan Ding, Hanyu Liu, Junyu Chen, Anjali P. Kusumbe

**Affiliations:** 1 Tissue and Tumor Microenvironments Lab, Cancer Discovery and Regenerative Medicine Program, Lee Kong Chian School of Medicine, Nanyang Technological University, Singapore 636921, Singapore; 2 Multidisciplinary Institute of Ageing (MIA-Portugal), University of Coimbra, 3004-504 Coimbra, Portugal; 3 State Key Laboratory of Oral Diseases, National Center for Stomatology, National Clinical Research Center for Oral Diseases, Department of Head and Neck Oncology, West China Hospital of Stomatology, Sichuan University, Chengdu 610041, China; 4 State Key Laboratory of Oral Diseases, National Center for Stomatology, National Clinical Research Center for Oral Diseases, Department of Prosthodontics, West China Hospital of Stomatology, Sichuan University, Chengdu 610041, China

**Keywords:** Cell Biology, Microscopy, Biotechnology and bioengineering

## Abstract

High-resolution whole-organ imaging provides cellular and molecular insights into tissue and tumor microenvironments. Here, we present a protocol for processing whole soft tissue organs and human tissues for 3D imaging within 2–2.5 days. We describe steps for bleaching, antigen retrieval, and collagenase-based digestion, followed by whole-tissue immunolabeling. We then detail procedures for dehydration and optical clearing for imaging by light-sheet microscopy.

For complete details on the use and execution of this protocol, please refer to Biswas et al.^1^

## Highlights

• Detailed protocol for an ultrafast 3D immunolabeling and clearing method
• Method for whole-organ 3D visualization of mouse and human tissues
• Detailed protocol for preserving tissue architecture and endogenous fluorescence

## Abstract (editor highlights)

Publisher’s note: Undertaking any experimental protocol requires adherence to local institutional guidelines for laboratory safety and ethics.

## Graphical abstract

**Figure:**
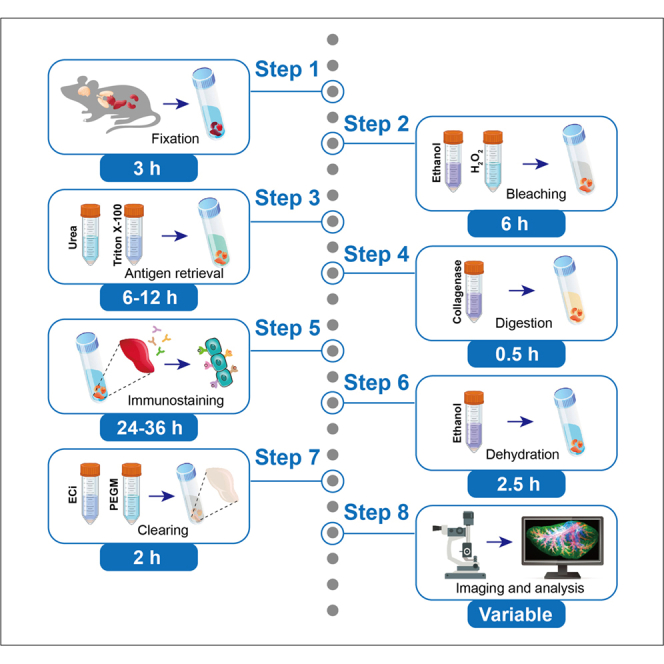

## Before you begin

Advanced three-dimensional (3D) imaging approaches provide critical insights into the fundamentals of highly complex biological processes during organ development, regeneration, repair and pathology.^2^^,^^3^^,^^4^^,^^5^^,^^6^^,^^7^^,^^8^^,^^9^^,^^10^ However, the acquisition of these high-content datasets typically comes with significant challenges, including extensive sample preparation, long processing times, large data volumes, and analytical complexity.^11^^,^^12^^,^^13^^,^^14^^,^^15^^,^^16^ In this paper, we describe our experimental protocols for the method we overcome these difficulties. We performed an ultrafast immunolabeling and solvent-based optical clearing of intact organs using light-sheet fluorescence microscopy, as performed in our associated study to visualize blood and lymphatic vasculature in mouse and human organs.^1^ It is worth mentioning that there are a few changes and adjustments in this protocol, which applies to soft organs and tissues only. For example, we removed the decalcification step used in our previous bone study, and incubation times were shortened because soft organs have better permeability compared to bones. Other procedures were also modified due to the different nature of soft organs from calcified tissues, such as the dehydrating agent. The protocol can also be applied to other murine organs and selected human tissue samples if proper antibodies are available, and processing time might be adjusted according to sample size or other properties. Specifically, as for organs with an abundant blood supply, cardiac perfusion is recommended to remove excessive hemosiderin, and the bleaching can be prolonged to acquire better tissue transparency. When the samples with a big volume are processed, it takes a longer time to incubate the sample for immunostaining and clearing. This rapid and reproducible approach enables high-resolution whole-organ imaging for the analysis of complex tissue architecture and rare cell populations.

### Institutional permissions

All the animals utilized for the studies were housed at Nanyang Technological University and University of Oxford. The experimental procedures on mice were performed following the UK Home Office Animal Care guidelines on the Operation of the Animals (Scientific Procedures) Act 1986 and the protocols approved by the local Animal Welfare and Ethical Review Board and by the UK Government Home Office (Animals Scientific Procedures Group). The sample of carcinoma of the buccal mucosa was obtained from the West China Hospital of Stomatology under the local EC-approved protocols at Sichuan University following informed consent from a 63-year-old female patient.

### Innovation

This protocol enables rapid tissue clearing and whole-organ immunostaining within 2 to 2.5 days, providing a robust method for acquiring high-resolution 3D images and investigating tissue microenvironments at the whole-organ level. As a methodological innovation, this protocol adopts several pivotal steps, including bleaching, antigen retrieval, and collagenase-based digestion, to achieve optimal tissue transparency and efficient immunolabeling. Light-sheet microscopy can be used to obtain high-resolution 3D images, and Imaris software is a powerful tool for advanced analysis and quantitative statistics. In view of the advantages above, this protocol offers significant improvements over previous methods, and it is broadly applicable to biomedical research.

## Key resources table

**Table:** 

REAGENT or RESOURCE	SOURCE	IDENTIFIER
**Chemicals, peptides, and recombinant proteins**		

Paraformaldehyde (PFA)	Sigma-Aldrich	P6148
Collagenase A	Merk	10103578001
Dimethyl sulfoxide	Sigma-Aldrich	D5879
Donkey serum	Abcam	ab7475
Ethyl cinnamate	Sigma-Aldrich	112372
Evans Blue	Sigma-Aldrich	E2129
Fetal bovine serum	Sigma-Aldrich	F7524
Glutaraldehyde	Sigma-Aldrich	340855
Glycerol	VWR	24388.260
30% H_2_O_2_	Sigma-Aldrich	H1009
Ethanol	Sigma-Aldrich	1070172511
Paraformaldehyde	Sigma-Aldrich	P6148
DMSO	Sigma-Aldrich	D5879
PBS	VWR	437117 K
poly(ethylene glycol) methyl ether methacrylate (PEGM)	Sigma-Aldrich	447943
Tamoxifen	Sigma-Aldrich	T5648
Triton X-100	Sigma-Aldrich	T8787
Urea	VWR	28876.367

**Antibodies**		

VEGFR2	Abcam	ab11939
α-SMA	Sigma-Aldrich	C6198
Endoglin	R&D Systems	AF1320
CD8⍺	Abcam	ab237368
LYVE1	Abcam	ab14917
Endomucin	Santa Cruz Biotechnology	sc-65495
Donkey anti-Rat IgG Alexa Fluor 594	Thermo Fisher Scientific	A21209
Donkey anti-Goat IgG Alexa Fluor 488	Thermo Fisher Scientific	A11055
Donkey anti-Goat IgG Alexa Fluor 647	Thermo Fisher Scientific	A21447
Donkey anti-Goat IgG Alexa Fluor 546	Thermo Fisher Scientific	A-11056
Alexa Fluor 488 streptavidin conjugate	Thermo Fisher Scientific	S11223
Alexa Fluor 546 streptavidin conjugate	Thermo Fisher Scientific	S11225
Donkey anti-Rabbit IgG Alexa Fluor 488	Thermo Fisher Scientific	A21206
Donkey anti-Rabbit IgG Alexa Fluor 647	Thermo Fisher Scientific	A31573

**Software and algorithms**		

Imaris	Oxford Instructment	https://imaris.oxinst.com/packages

**Other**		

Shaking water bath	Stuart	SBS40
Lightsheet microscope	Miltenyi-LaVision Biotech

**CRITICAL:** PFA and glutaraldehyde are toxic. The fixative solution should be prepared inside a fume hood.

### Light-sheet microscope setup

a. Equip with a 2X objective lens for a zoom body with a manual zoom of 0.63X– 6.3X.
b. Fit with a Dipping Cap [5.7 mm] including Optics for Olympus MVPLAPO 2X.
c. The 405 nm-100 mW, 488 nm-85 mW, 561 nm-100 mW, 639 nm-70 mW and 785 nm-75 mW laser lines and the images captured with the Neo sCMOS camera (Andor).

## Materials and equipment

**Table:** Fixation buffer

REAGENT	Final concentration	Amount
paraformaldehyde	4%	4 g
glutaraldehyde	0.05%	50 μL
PBS	1X	99.95 mL

Stored at 20 °C for up to 4 weeks.

**CRITICAL:** PFA and glutaraldehyde are highly toxic and should be manipulated in the fume hood, and the appropriate PPE is required.

**Table:** Bleaching buffer

REAGENT	Final concentration	Amount
Ethanol	95%	95 mL
30% H_2_O_2_	1.5%	5 mL

Prepare fresh each time.

**Table:** Antigen Retriever Solution

REAGENT	Final concentration	Amount
urea	25 weight%	25 g
glycerol	15%	15 mL
Triton-X100	7%	15 mL
ddH_2_O	–	70 mL

Make up fresh each time and dispose of any unused solution properly.

**Table:** Digestion buffer

REAGENT	Final concentration	Amount
Collagenase A	0.2%	0.2 g
PBS	1X	100 mL

**Table:** Washing buffer 1

REAGENT	Final concentration	Amount
FBS	2%	400 μL
PBS	1X	19.6 mL

**Table:** Blocking buffer

REAGENT	Final concentration	Amount
donkey serum	10%	1 mL
DMSO	10%	1 mL
Triton X-100	0.5%	50 μL
PBS	1X	8 mL

Prepare fresh each time.

**Table:** Antibody buffer

REAGENT	Final concentration	Amount
donkey serum	2%	20 μL
DMSO	10%	100 μL
Triton X-100	0.5%	5 μL
Antibodies	1:500	2 μL
PBS	1X	900 μL

**Table:** Antibody dilutions

ANTIBODY	Dilution
VEGFR2	1:300
α-SMA	1:200
Endoglin	1:300
CD8⍺	1:300
LYVE1	1:200
Endomucin	1:300
Donkey anti-Rat IgG Alexa Fluor 594	1:500
Donkey anti-Goat IgG Alexa Fluor 488	1:500
Donkey anti-Goat IgG Alexa Fluor 647	1:500
Donkey anti-Goat IgG Alexa Fluor 546	1:500
Alexa Fluor 488 streptavidin conjugate	1:500
Alexa Fluor 546 streptavidin conjugate	1:500
Donkey anti-Rabbit IgG Alexa Fluor 488	1:500
Donkey anti-Rabbit IgG Alexa Fluor 647	1:500

**Table:** Washing buffer 2

REAGENT	Final concentration	Amount
donkey serum	2%	20 mL
Triton X-100	0.5%	5 mL
PBS	1X	1000 mL

**Table:** Clearing buffer

REAGENT	Final concentration	Amount
ECi	80%	80 mL
Poly (ethylene glycol) methyl ether methacrylate (PEGM)	20%	20 mL

Prepare fresh each time.

## Step-by-step method details

The procedure is described according to the processing of mouse soft organs, so the incubation or digestion times should be adjusted when applied to the samples from other animals or humans in view of sample size differences. For example, the digestion time should be prolonged since the human samples have stronger and denser extracellular matrix, which impedes the permeabilization of the antibody. The volume of all processing solutions below should be at least ten times that of the samples.

### Tissue collection and fixation

**Timing: 3 h and 15 min (for steps 1–3)**

1. Rinse freshly dissected tissues in PBS for seconds.

***Note:*** All excessed muscles and fats are recommended to be removed before fixing are recommended. Cardiac perfusion is recommended, especially for the organs with an abundant blood supply, such as the heart, spleen, and kidney. This step can remove excessive hemosiderin and thus acquire better tissue transparency. While the omission of perfusion does not significantly impact the imaging of other light-colored samples as demonstrated by our results. Overall, cardiac perfusion does not affect the efficiency of immunolabeling; instead, it is beneficial to improve the tissue transparency by removing excessive hemosiderin, which is positively related to imaging depth and quality.

**Figure 1:**
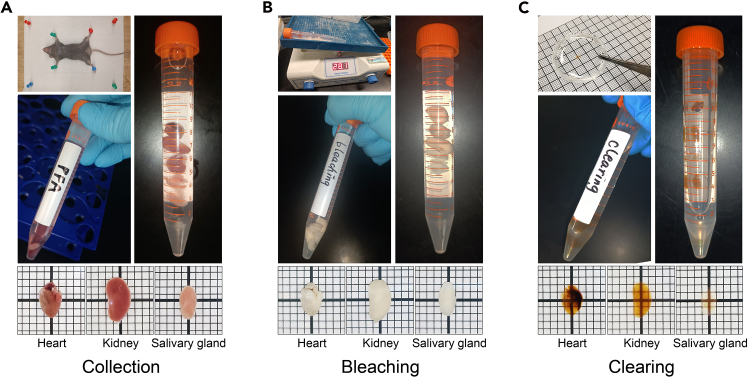
The operation process of the main steps and the photos of the sample in different stages (A) The collection of mouse organs and the fixation of the sample with PFA. (B) The bleaching of the samples with ethanol and H2O2 on a shaker. The organs turn pale after bleaching. (C) The clearing of the samples in the clearing medium containing ECi and PEGM. The organs turn transparent after clearing.

***Note:*** The volume of fixative solution should be at least ten times that of the samples to ensure proper fixation. Moreover, tubes with a flat bottom are recommended to ensure that the samples are immersed in the solution.

2. Immediately transfer tissues into fixative solution at 4°C for 3 h on a shaker. (see Figure 1A). To confirm that the impacts of fixation time on the immunostaining and imaging, we have supplemented different images to show the imaging outcomes of different processes (0.5 h, 2 h, 3 h, 6 h, 10 h), which demonstrate that the 3 h group presents the optimal immunostaining and imaging quality (see Figure S1A), so we emphasize the 3 h of incubation time here.

3. Wash the samples with PBS for 3 times at 25°C on a shaker. Each wash lasted for 5 min.

***Note:*** The fixed samples can be stored for up to 4 days at 4°C before moving forward with the bleaching or antigen retrieval and permeabilization. The volume of PBS should be more than the fixative solution to completely remove the fixative solution.

### Bleaching

**Timing: 6 h (for steps 4–6)**

4. Immerse samples in ethanol gradient of 50%, 80% and 100% for 30 min each for dehydration. 100% ethanol was changed twice, 30 min apart.

***Note:*** These dehydration buffers should be 4°C. The bleaching reagent is H_2_O_2_, and ethanol serves as the medium carrying H_2_O_2_. Given that bleaching is conducted after dehydration, the use of an aqueous solution will lead to obvious tissue swelling and deformation. Besides, ethanol permeates into the tissue more easily and improves bleaching efficiency. Therefore, we bleached the sample in ethanol after dehydration, and then rehydrated the sample.

5. Immerse samples in 5% (v/v) H_2_O_2_ in ethanol for up to 3 h for bleaching (see Figure 1B).

***Note:*** During bleaching, the samples should be kept at 4°C and avoid light.

6. Immerse organs in an ethanol gradient of 100%, 80% and 50% for 30 min each for dehydration.

### Antigen retrieval

**Timing: 6–12 h (for step 7)**

7. Incubate tissues with antigen retrieval buffer at 4°C for 6**–**12 h.

***Note:*** The time of antigen retrieval depends on the sample size and tissue density. 6 h are enough for small mouse organs, such as the adrenal gland; instead, big organs as big as the heart and even the mouse brain need a longer time up to 12 h. We list the major organs of the mouse and the corresponding incubation time for readers to refer to.

**Table:** 

Organs	Incubation time of antigen retrieval
Adrenal gland	6 h
Ovary	6 h
Lung	6 h
Parotid gland	7 h
Thyroid gland	7 h
Lacrimal gland	7 h
Lymph node	7 h
Thymus	8 h
Submandibular gland	8 h
Gut	8 h
Skin	8 h
Testis	9 h
Liver	9 h
Spleen	9 h
Bladder	10 h
Tongue	10 h
Kidney	10 h
Heart	12 h
Brain	12 h

### Collagenase digestion

**Timing: 40 min (for steps 8 and 9)**

8. Directly incubate samples with the digestion solution for up to 30 min at 37 °C on constant shaking.

***Note:*** The time of digestion should be limited to 30 min to avoid damage to tissue integrity and ensure proper permeability of the samples. To ensure that cell markers are maintained after the process, we have done experiments to test the integrity and immunogenicity of the samples using a gradient of collagenase and time of incubation. Based on our results and experience, we selected 0.2% collagenase as the optimal concentration and incubated the samples for 30 min.

9. Wash samples twice for 5 min with the washing buffer 1.

### Immunolabeling

**Timing: 24–36 h (for steps 10–14)**

The time of incubation depends on the sample size and antibody concentrations, which needs to be explored for beginners. We list the major organs of the mouse and the corresponding incubation time for readers to refer to.

**Table:** 

Organs	Incubation time of primary antibody	Incubation time of secondary antibody
Adrenal gland	14 h	6 h
Ovary	14 h	6 h
Lung	14 h	6 h
Parotid gland	14 h	6 h
Thyroid gland	14 h	6 h
Lacrimal gland	14 h	6 h
Lymph node	14 h	6 h
Thymus	15 h	7 h
Submandibular gland	15 h	7 h
Gut	15 h	7 h
Skin	15 h	7 h
Testis	15 h	7 h
Liver	15 h	7 h
Spleen	16 h	8 h
Bladder	16 h	8 h
Tongue	16 h	8 h
Kidney	16 h	8 h
Heart	16 h	8 h
Brain	16 h	8 h

10. Incubate samples in blocking solutions at 37 °C for 20 min in a shaking water bath rotating at 70 rpm.
11. Incubate samples with Primary antibody solution for 14 to 16 h at 37 °C in a shaking water bath rotating at 70 rpm with the primary antibodies at a dilution of 1:300.
12. Wash samples with washing buffer 2 for 3 h at 37 °C in the shaking water bath rotating at 70 rpm. The wash buffer was changed every 15 min for the first hour and then every 30 min for the remaining 2 h.
13. Incubate samples in secondary antibodies buffer at a dilution of 1:500 for 6 to 8 h at 37 °C in the shaking water bath rotating at 70 rpm.
14. Wash samples with washing buffer 2 for 3 h with the same procedure above at 37 °C in the shaking water bath rotating at 70 rpm.

### Dehydration

**Timing: 2.5 h (for steps 15–16)**

15. Immerse samples in a gradient of 30%, 50% and 80% ethanol for 30 min each under gentle rotation at 25°C.
16. Immerse samples in 100% ethanol for 1 hour with the 2 changes of ethanol during this duration.

### Clearing

**Timing: 2 h (for steps 17–19)**

17. Completely remove ethanol.
18. Rinse samples twice with ethyl cinnamate (ECi) for 10 min each.

***Note:*** ECi is harmful/irritant and should be handled in the fume hood, and the appropriate PPE is required.

19. Cleared samples with 80% ECi and 20% poly(ethylene glycol) methyl ether methacrylate (PEGM) under gentle rotation at 25°C for 100 min (see Figure 1C).

### Imaging

**Timing: Various (for step 20)**

20. Lightsheet Fluorescent Microscope (Lightsheet 7 ZEISS)
  a. Mount the samples and attach them to the sample holder using superglue.
  b. Fill the tank with ECi (refractive index=1.57).
  c. In the maintenance section, inform the software about the objective you selected.
  d. Place the sample holder on the platform. We take the kidney as an example to show readers how we fix the sample on the holder. The sample is fixed on the holder with glue, and the holder is placed in the imaging chamber filled with ECi (see Figure S1B).
  e. Position the sample in front of the objectives. The illumination objectives include LSFM 5X/0.1 foc and LSFM 10X/0.2 foc. The detection objectives include EC Plan-Neofluar 5X/0.16 foc (working distance =10.5 mm) and Clr Plan-Neofluar 20X/1.0 Corr (working distance =6.4 mm). Adjust illumination objective and detection objectives according to the refractive index of the imaging medium (Eci). The pixel size of the kidney image is 0.92x0.92x3.71 μm.
  f. Adjust the power of the laser (10**–**20%), stepsize (2**–**4), thickness (3**–**6 μm), wavelengths (405, 488, 561, 639, and 785), and exposure time (10 μs-100 ms) according to specific situations and imaging requirements to achieve the maximal signal and lowest background noise. The excitation and emission wavelengths of the dyes we used are listed below.

**Table:** 

Dye	Excitation wavelength (nm)	Emission wavelength (nm)
DAPI	364	454
FITC	492	520
CY3	550	570
CY5	648	662
  g. Define the upper and lower sample stacks to determine the z-volume to be imaged, which depends on the thickness of the sample or the area of interest. For example, a kidney sample contains about 600**–**800 layers.
  h. Start the acquisition and save the raw data.

### Data analysis

**Timing: Various (for steps 21 and 22)**

**Figure 2:**
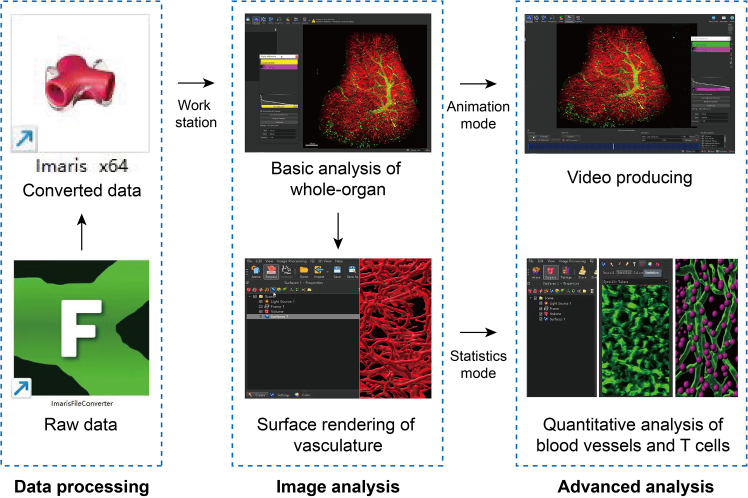
Pipeline for data processing and image analysis Graphical pipeline illustrating the key steps of data processing, basic image analysis, and advanced data analysis. Image analysis contains basic 3D image acquisition and surface rendering. Advanced analysis involves animation and quantification.

21. Convert raw image data using the Imaris File Converter (Bitplane).
22. Analyze the converted data using Imaris software (version 9.9.0, Bitplane).
  a. Utilize Imaris software to reconstruct and process the Z-sections of the light-sheet images, and define the Regions of Interest (ROI) for individual channels of certain tissue architectures, and then reconstruct the surface.
  b. Set the resolution for different vessels or spots. Employ surface analysis XTensions tools within Imaris to perform vascular density quantification. Utilize the 3D crop tool to analyze the total tissue volume. Smooth the ROI post-surface reconstruction and subtract the background using appropriate settings, and preview the final images before being generated.
  c. Employ advanced tools of Imaris software to achieve animation displaying and quantitative analysis, facilitating comprehensive scientific insights.
  d. Conduct quantitative analysis cted based on the surface rendering and ROI definition. The open-source software, such as ImageJ, is able to perform basic data processing, but not advanced analyses, including surface and spot rendering and animation. Thus, we recommend Imaris as the optimal data analysis software (see Figure 2).

## Expected outcomes

**Figure 3:**
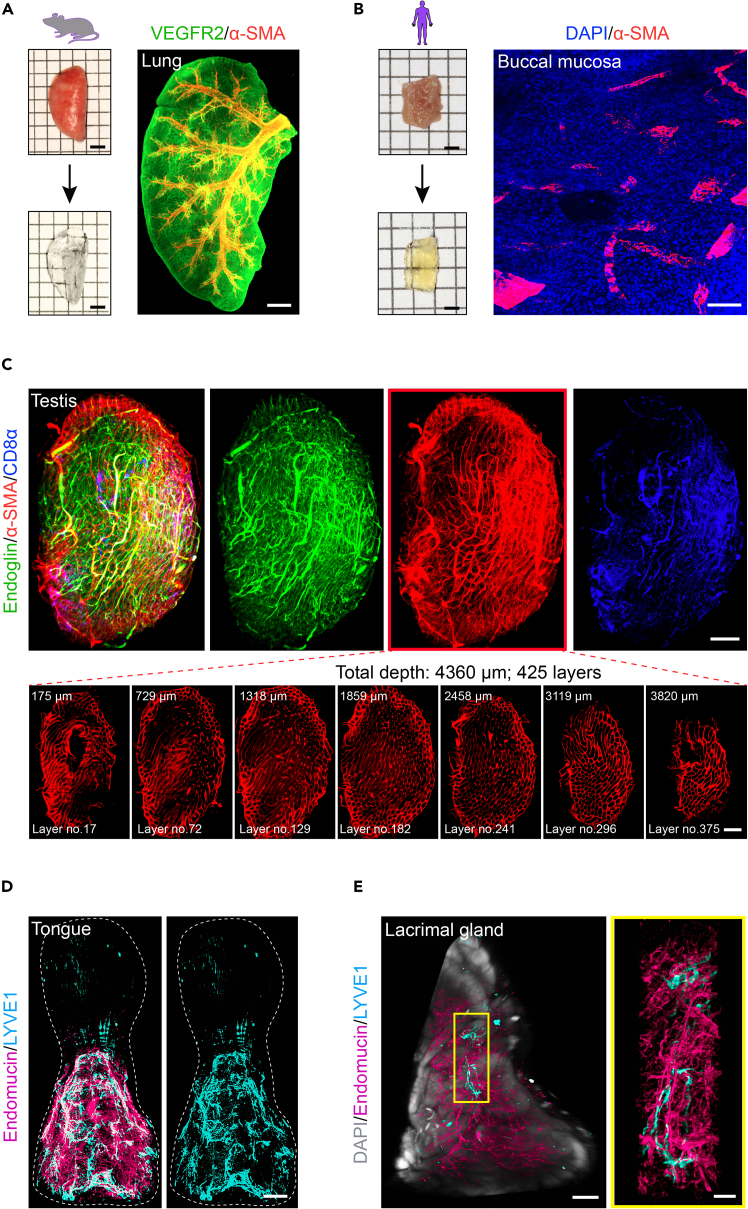
Multicolor panoptic light-sheet imaging of mouse and human samples with immunostaining and endogenous fluorescence (A) Images of a mouse lung before and after tissue clearing. 3D light-sheet image of a mouse lung immunostained with VEGFR2 and α-SMA. Grid scale: 500 μm, Scale bars: 500 μm. (B) Images of the buccal mucosa from an oral cancer patient before and after tissue clearing. Representative image of the transparent human buccal mucosa labeled by DAPI and α-SMA. Grid scale: 600 μm, Scale bars: 500 μm. (C) Panoptic light-sheet image of an intact mouse testis immunolabeled by Endoglin, α-SMA, and CD8α. The slice views gallery shows the representative longitudinal sections across the entire testis with a total depth of 4360 μm. Scale bars: 400 μm. (D) Light-sheet image of a whole mouse tongue labeled by DAPI and immunostained with Endomucin and LYVE1. Scale bars: 600 μm. (E) Light-sheet image of a whole mouse lacrimal gland labeled by DAPI and immunostained with Endomucin and LYVE1. Scale bars: 400 μm, inset 100 μm.

Using this protocol, we have achieved tissue transparency across multiple murine organs and human tissue in the shortest possible time. When processing fatty tissues, we recommend extending the antigen retrieval time to 12 h to improve tissue permeability, and prolonging the immunostaining time to 36 h to ensure thorough labeling. There are no additional steps in these cases, and we do not need to carry out delipidation in our protocol, while we have to dissect away the fatty tissues around the sample. The above procedure applies to basically all kinds of murine organs, including the heart, kidney, and brain. The protocol might need to be adjusted when used for bigger samples than 2 cm, such as prolonging the time and elevation of reagent concentrations. Multicolour immunolabeling together with light-sheet microscopy has enabled excellent 3D tissue morphology and architecture (see Figures 3A and 3B). We have successfully validated this method across multiple murine organs, including lung, testis, tongue, and lacrimal gland, allowing clear visualization of the 3D architecture of immunolabeled vascular and lymphatic networks throughout intact organs (see Figures 3A–3E). Moreover, this protocol enables deep tissue immunolabeling of intact whole organs, and the immunolabeled signals remain uniform along the entire depth of the organ (see Figure 3C). To distinguish true blood vessel staining from tissue autofluorescence, we performed an experiment including a negative control without primary antibody, which displays the real signals of LYVE1-positive lymphatic endothelia in the whole mouse submandibular gland (see Figure S1C). We also provide representative 3D imaging videos highlighting spatial organization and cellular interactions within intact organs (see Methods Video S1).

Document S1. Figure S1

## Limitations

This protocol is readily applicable to intact, unsectioned soft tissues. All the utilized reagents are commonly available in biomedical research laboratories. However, a notable limitation of this protocol is its reliance on lightsheet microscopy. This demand for unique equipment may limit the adoption of this protocol in certain research settings. Further, the resolution of the Z-axis is limited due to the nature of light-sheet microscopy, which is the reason why tissue sectioning and confocal imaging are still irreplaceable, despite whole-organ imaging has come to being.

## Troubleshooting

### Problem 1

High background along with the antibody signal due to the opaqueness and hemosiderin.

### Potential solution

• Bleaching is required to remove pigmentation and reduce hematoma signal, and cardiac perfusion is critical for the organs with an abundant blood supply to remove as much hemosiderin as possible (Step 5).
• Optimised dehydration as suggested on the protocol. Specifically, samples should be submerged in the ethanol, and the time of each gradient dehydration ought to be 30 min at least and up to 40 min (Steps 4 and 5).

### Problem 2

Strong signal at the tissue surface with weak internal labeling.

### Potential solution

• Extend the time of digestion, but not longer than 30 min. The digestion ought to be at 37 °C on constant shaking to ensure the efficacy of the digestion (Step 8).

### Problem 3

Strong signal at the tissue surface with weak internal labeling.

### Potential solution

• Extend the time of antibody incubation and ensure proper shaking at 37 °C. The incubation time of primary and secondary antibodies can be extended to 24 h and 12 h, respectively (Steps 11 and 13).

### Problem 4

Weak antibody signal.

### Potential solution

• Antibody concentration is inadequate to stain the whole organ. Increasing the antibody concentration will increase the signal, especially for the primary antibody; the concentration can be increased to 1/200 from 1/300 (Steps 11 and 13).

### Problem 5

Antibodies precipitations on the organs.

### Potential solution

• Ensure adequate washing is carried out to overcome this issue. The washing ought to last for 3 h at 37 °C in the shaking water bath rotating at 70 rpm, and the washing buffer can be changed more frequently if needed (Steps 12 and 14).

### Problem 6

High background along with antibody signal.

### Potential solution

• Wash more times can remove excessive non-specific fluorescence (Steps 12 and 14).

### Problem 7

Noisy and non-transparent fluorescence signal.

### Potential solution

• Ensure the dehydration is enough to get a transparent signal as per protocol. Specifically, samples should be submerged in the ethanol, and the time of each gradient dehydration ought to be 30 min at least and up to 40 min. Notably, the dehydration of 100% ethanol must be conducted twice to ensure complete dehydration (Steps 15 and 16).

### Problem 8

Bubbles build up inside the whole organ.

### Potential solution

• Fill the space in the organ with clearing solution by needle without damaging the tissues, avoiding refraction background within the images (Step 19).

## Resource availability

### Lead contact

Further information and requests for resources and reagents should be directed to and will be fulfilled by the lead contact, Anjali P. Kusumbe (anjali.pkusumbe@ntu.edu.sg).

### Technical contact

Questions about the technical specifics of performing the protocol should be directed to the technical contact, Junyu Chen (junyuchen@scu.edu.cn).

### Materials availability

This study did not generate new unique reagents.

### Data and code availability

This study did not generate or analyze datasets. This study did not report original code.

## Acknowledgments

A.P.K. is supported by the 10.13039/501100001459Ministry of Education (MOE), Singapore: Academic Research Funds (#024983-00001 and #025277-00026), the European Research Council (StG: metaNiche, 805201), and the European Union’s Horizon 2020 (no 857524). J.C. is supported by the National Nature Science Foundations of China (nos. 82270961 and 82422021).

## Author contributions

Z.D. and H.L.: methodology, investigation, visualization, and writing. J.C.: methodology, validation, and formal analysis. A.P.K.: supervision, reviewing, and editing.

## Declaration of interests

The authors declare no competing interests.

## Supplemental information

Methods Video S1. Lung processed by this method showing multicolor immunostaining for CD31-positive endothelial blue, α-SMA-positive (red) and VEGFR2-positive green perivascular cells, related to Step 22
