# Supplementary material for: Protocol for ultrafast immunolabeling and 3D imaging of whole organs and large tissues
Source: STAR Protoc. 2026 Jun 6;7(2):104623. doi: 10.1016/j.xpro.2026.104623 (PMC13260106; doi:10.1016/j.xpro.2026.104623)
Supplement: Document S1. Figure S1 [file mmc1.pdf]

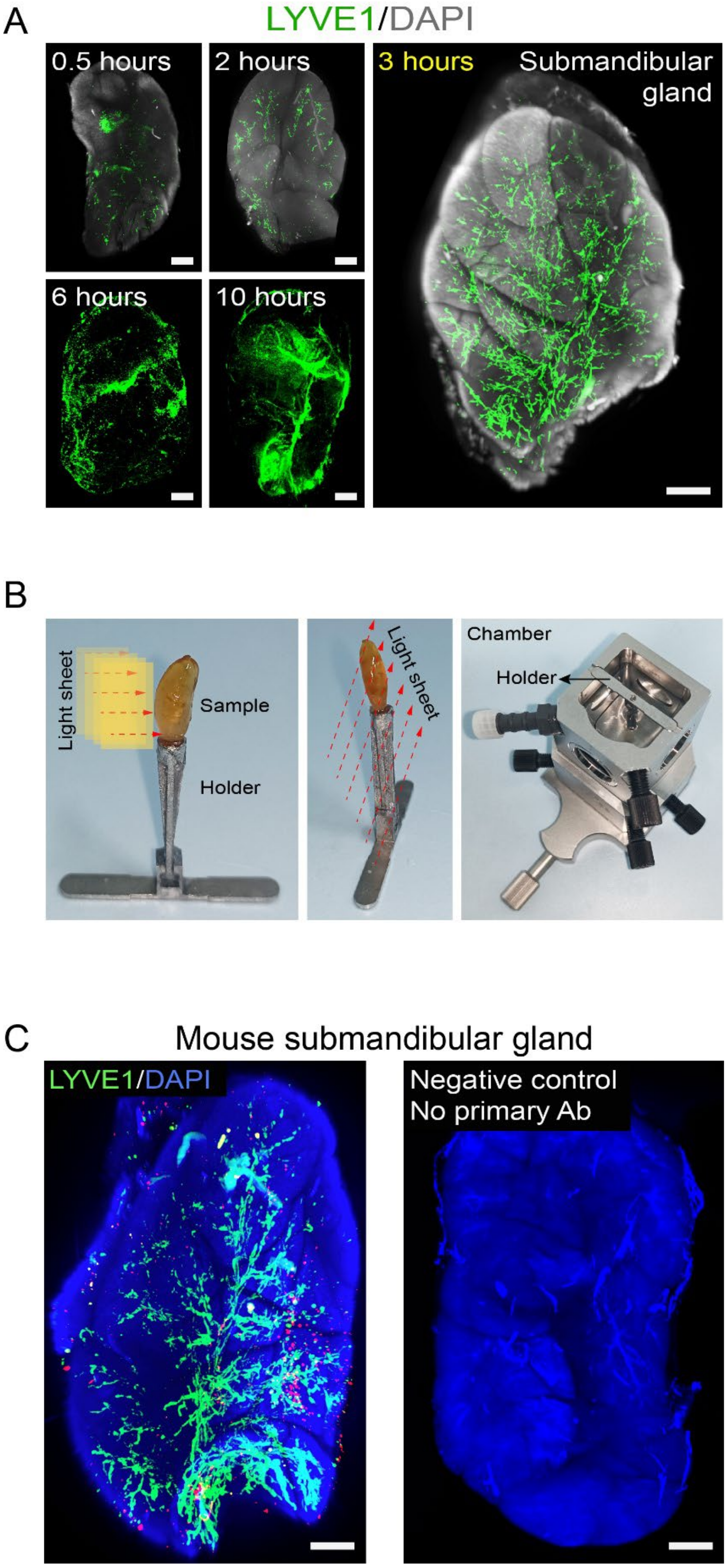

Figure S1: Comparison of imaging results demonstrating negative control and various fixation time groups, related to Step 2 and Step 20.

(A) Representative images of the samples fixed for various times and labeled by LYVE1 (green) and DAPI (grey). Scale bars: 500 μm.

(B) Sample glued on the holder in a proper orientation and transferred to the imaging chamber filled with ECi.

(C) In the negative control, primary antibody was not added and the sample was stained only with secondary antibodies and DAPI. Alexa Fluor 488 (green), Alexa Fluor 405 (blue). Scale bars: 500 μm.
